# Gender Differences in Depressive Symptomatology Among Turkish Immigrants: An Analysis of PHQ9 Scores

**DOI:** 10.1192/j.eurpsy.2025.1312

**Published:** 2025-08-26

**Authors:** E. D. Cindik-Herbrueggen

**Affiliations:** 1 Psychiatry, Neuro-Psychiatric Center, München, Germany

## Abstract

**Introduction:**

Depression is a pervasive mental health disorder that can significantly influence symptomatology, which remains a crucial area of research. This study aims to investigate whether gender correlates with PHQ-9 total scores, enhancing the general understanding of depression across different demographic groups.

**Objectives:**

This study examines the correlation between gender and PHQ-9 total scores, investigating the hypothesis that gender influences various depressive symptoms as measured by the PHQ-9. We aim to determine whether there is a significant difference in depression severity between male and female participants.

**Methods:**

To fulfill this purpose, we collected data from a total of 146 participants, primarily Turkish immigrants of the first and second generation, who were recruited from the Neuro-Psychiatrisches Zentrum Riem. First, we gathered the participants’ sociodemographic data, including gender, marital status, and education level. Furthermore, they were asked to complete the PHQ-9 questionnaire, and we conducted a Pearson correlation analysis.

**Results:**

The PHQ-9 total scores and gender show a significant correlation, with a p-value of 0.040. Furthermore, these results indicate that female participants had higher average PHQ-9 scores compared to male participants, suggesting a greater intensity of depressive symptoms among women. Since the participants were predominantly Turkish immigrants, these findings may suggest that Turkish women are more prone to severe depressive symptoms, potentially as a consequence of their immigration history.

**Conclusions:**

This study emphasizes the significance of gender in relation to depressive symptoms, as measured by the PHQ-9 questionnaire. However, future research should investigate other factors, besides gender, that may influence the severity of depressive symptoms in immigrant participants. It is recommended that these studies focus on the long-term effects of immigration, depression, and gender by including a diverse range of participants, such as immigrants from the first, second, third, and fourth generations. These generational differences could indicate significant variations in the experiences and consequences of severe depressive symptoms.

**Disclosure of Interest:**

None Declared

